# Evaluation of the presence of gingivitis as confounding factor in assessing inflammatory status in serum and saliva of dogs with diabetes mellitus

**DOI:** 10.1186/s12917-024-03962-8

**Published:** 2024-03-23

**Authors:** Lorena Franco-Martinez, Alberto Muñoz-Prieto, Francesca Busato, Birute Karveliene, Inga Stadaliene, Jose J. Ceron, Juana D. Carrillo, Juan D. Garcia-Martinez, Roman Dabrowski, Luis Pardo-Marín, Silvia Martinez-Subiela, Asta Tvarijonaviciute

**Affiliations:** 1 Interdisciplinary Laboratory of Clinical Analysis, Interlab-UMU, Regional Campus of International Excellence “Campus Mare Nostrum”, University of Murcia, Ed. 16, Espinardo, Murcia 30100 Spain; 2 Division of Internal Medicine, San Marco Veterinary Clinic, Veggiano, PD Italy; 3 Dr. L. Kriaučeliūnas Small Animals Clinic, Faculty of Veterinary, Veterinary Academy, Lithuanian University of Health Sciences, Tilžės str. 18, Kaunas, 47181 Lithuania; 4 Department of Animal Medicine and Surgery, Faculty of Veterinary Medicine, Regional Campus of International Excellence “Campus Mare Nostrum”, University of Murcia, Murcia, Spain; 5 Department and Clinic of Animal Reproduction, Faculty of Veterinary Medicine, University of Life Sciences, Gleboka 30, Lublin, 20-612 Poland

**Keywords:** Canine, Diabetes mellitus, Gingivitis, Interleukins, Saliva

## Abstract

The aim of this study was to evaluate the changes in the serum and salivary inflammatory markers induced by Diabetes mellitus (DM) in dogs and to assess the possible confounding effect of gingivitis. A panel of 13 cytokines was measured in the serum and saliva of dogs diagnosed with DM and compared with healthy dogs without gingivitis (control group 1; CG1) and dogs with gingivitis but otherwise healthy (control group 2; CG2). The results of the present study showed statistically significantly higher levels of IL-8, KC-like and MCP1 in the serum of dogs with DM compared to CG1 dogs. In the case of saliva, the DM group presented statistically higher GM-CSF, IL6, IL15, and MCP1 levels compared to CG1, and lower KC-like chemokine compared to CG2. Finally, gingivitis produced changes in saliva, with salivary levels of GM-CSF, IL-6, IL-7, IL-15, IP-10, KC-like, IL-10, IL-18, MCP1, TNFα being statistically significantly higher in the saliva of CG2 dogs compared to CG1. The results of the present study indicate that dogs with DM have altered cytokine levels in serum and saliva compared to healthy dogs. In addition, this study highlights the importance of taking oral health into account when determining cytokines in dogs, as gingivitis can significantly alter their concentrations. .

## Introduction

Diabetes mellitus (DM) is one of the most common endocrine disorders in dogs with a prevalence that can reach 1.5% [1]. Canine DM is currently diagnosed by observation of compatible clinical signs such as polyuria, polydipsia and polyphagia and confirmed by detection of persistently increased glucose in the blood.

Saliva is a non-invasive sample increasingly used in human and veterinary medicine. Markers of stress such as cortisol, inflammation such as C-reactive protein, and kidney function such as urea and creatinine, have been measured in the saliva of dogs showing a positive correlation with their concentrations in serum [2–4]. In humans, DM is related to the presence of systemic inflammation, therefore, inflammatory biomarkers were studied in the saliva of patients with DM by different authors [5–7]. Nevertheless, oral inflammation is considered one of the main confounding factors for the accurate determination and interpretation of salivary biomarkers in systemic diseases [8]. In the case of severe gingivitis, blood leakage could significantly alter results since the concentrations of some of the components in the blood can be around 1000 fold higher than in saliva [2]. Therefore, caution should be taken to avoid errors in data interpretation in these situations. This is particularly important in DM since permanently increased circulating glucose levels can cause gingival inflammation due to altered vascular permeability and resistance to bacterial plaque impairment [9]. In dogs, severe gingivitis has been associated with increased lactate dehydrogenase (LDH) and phosphorus in saliva [10].

The hypothesis of this study was that inflammatory biomarkers could change in the saliva of dogs with canine DM and that also these biomarkers could be affected by gingivitis. Therefore, this work aimed to assess a panel of cytokines in the saliva and serum of dogs diagnosed with DM and to compare their concentrations in the saliva of healthy dogs with and without gingivitis.

## Materials and methods

### Ethical approval

The study complies with ***3R principles*** (replacement, reduction and refinement) in animal research, giving a special care to avoid any discomfort, suffering or pain in the patients. The protocols were approved by the Ethical Committee of the University of Murcia and Counseling of the Murcia Region, Spain (A13170503).

### Animals

A total of 61 dogs were included in the present study. All dogs were privately owned dogs, presented to private veterinary clinics of Murcia region, Spain, Veterinary Hospital “San Marco”, Padova, Italy, and L. Kriaučeliūnas Small Animal Clinic, Kaunas, Lituania, between June 2018 and June 2022. All animals were assigned to one of the following groups:

Control group consisted of a total of 45 healthy adult dogs that did not present abnormalities at physical examination, with the exception of gingivitis, nor in the routine CBC and biochemical profile. Twenty dogs were mongrels, six Yorkshire terriers, three Labrador Retrievers, three German Shepherds, two of each Maltese Bichon, Chihuahua, Pomeranian, and Dachshund, and one of each Border Collie, French Bulldog, Bull Terrier, Poodle, and Brazilian Fila. According to their gingival health status, control dogs were further divided into: Control Group 1 (CG1), healthy dogs with healthy gingiva (gingival health score = 0; *n* = 22) and Control Group 2 (CG2), dogs with gingivitis, otherwise healthy (gingival health score  = 1–3; *n* = 23).

The DM group consisted of 16 adult dogs diagnosed with canine DM. The diagnosis of DM was based on clinical signs and laboratory findings including polyuria, polydipsia, polyphagia, glucosuria and hyperglycaemia (glucose > 200 mg/dL) as previously described [11]. In all cases, a complete blood cell count and serum biochemical profile was performed to detect or exclude any concurrent disease; and only dogs with complete clinical data were enrolled in the study. Nine dogs were mongrels, and the resting were one of each - Poodle, Fox terrier, Rottweiler, Irish Setter, Siberian Husky, Weimaraner, and West Highland White Terrier.

In all dogs, the health status of gingiva was assessed based on a 4-point scale, where 0 = normal gingiva and 3 = severe inflammation [12]. Body condition score was considered using a 9-point scale [13].

### Biochemistry analysis

Saliva specimens were obtained as previously described [2]. In brief, a sponge was placed in dog’s mouth for 1–2 min and then passed into the Salivette (Salivette®, Sarstedt AG &Co., Nümbrecht, Germany) device for centrifugation (P Selecta®, JP Selecta S.A, Barcelona, Spain) at 3000 x g 10 min, 4 °C. Afterwards, saliva specimens were stored at -80 °C until cytokines were measured. Serum ,  surplus resting from the requested analysis by the responsible veterinary specialist was used.

Salivary and serum granulocyte macrophage-colony stimulating factor (GM-CSF), interferon gamma (IFN-γ), interleukin (IL) -2, IL-6, IL-7, IL-8, IL-10, IL-15, IL-18, interferon gamma-inducible protein 10 (IP-10), keratinocyte-derived chemokine-like (KC-like), monocyte chemoattractant protein 1 (MCP-1) and tumor necrosis factor alpha (TNF-α) were determined using commercially available assay (CCYTOMAG-90 K, MILLIPLEX MAP Canine Cytokine/Chemokine Magnetic Bead Panel - Immunology Multiplex Assay).

### Statistical analysis

All statistical analyses were performed using standard descriptive statistical procedures and software (GraphPad Prism Version 7 for Windows, GraphPad Software, La Jolla, CA; IBM SPSS Version 21, Enhingen, Germany). Since most data did not follow Gaussian distribution, nonparametric tests were used. Mann Whitney test was used to compare serum and salivary cytokines, age, body weight, body condition, and number of meals per day data between control dogs and dogs with DM, while the Chi-square test was employed to assess the statistical difference of the rest of descriptive variables between the two groups of dogs. When control dogs were subdivided into two groups according to gingival health status, Kruskal-Wallis test followed by Dunn`s multiple comparison test was employed. In all cases, the level of significance was set at *P* < 0.05.

## Results

Detailed descriptive data of dogs included in both groups are presented in Table 1. Animals included in the two groups of this study did not show statistically significant differences in terms of age, body weight, body condition score, or sex. In relation to feeding manners, groups did not differ in type of food offered, number of meals per day, or supplements given, while snack were more often included in the diet of healthy dogs (*P* < 0.001). Regarding exercise, there was statistically significant differences in both studied variables, type and time (*P* < 0.05 and *P* < 0.001, respectively).

**Table 1:** Descriptive data of control dogs and dogs with Diabetes mellitus (DM) included in present study

Variable	Controls (total) (*n* = 45)	DM (*n* = 16)	P
**Age, years**	9 (6.1–11.6)	10.0 (7.0–12.0)	0.525
**Body weight, kg**	10.8 (5.0–23.7)	13.0 (8.2–31.0)	0.342
**Body condition score**	5 (5–6)	5 (4–6)	0.239
**Sex**			0.477
Female	27 (60)	11 (68.8)	
Male	18 (40)	5 (31.2)	
**Gingiva health status**			**< 0.001**
Healthy (score = 0)	22	16	
Gingivitis (score = 1-3)	23	0	

Data of variables age, body weight, body condition score and number of meals per day are presented as median (25–75th percentile) and the difference between the groups were assessed using Mann Whitney test. The resting variables are presented as a number of patients (percent) and the difference between the groups were assessed using Chi-square. P-value in bold highlight statistical significance between the groups.

Dogs with DM compared to control dogs had 2.1-fold higher IL-6 (*P* < 0.05), 2.0-fold higher IL-8 (*P* < 0.05), 3-fold higher KC-like chemokine (*P* < 0.05) and 1.8-fold higher MCP1 (*P* < 0.01) concentrations in serum (Table 2).

**Table 2:** Median (25–75th percentile) data of serum levels of cytokines in dogs with Diabetes mellitus (*n* = 16) and controls (total, *n* = 45) with healthy gingiva (Control group 1 (CG1), *n* = 22) and with gingivitis (Control group 2 (CG2), *n* = 23)

	DM	Controls (total)	P*	CG1	CG2	P**
GM_CSF	14.5 (10.2–37.9)	16.8 (11.3–51.8)	0.522	15.1 (11.0-51.3)	25.3 (11.0-108.5)	0.763
IFNg	1.9 (1.7–2.2)	2.5 (2.0–7.0)	0.099	2.3 (1.8–3.5)	2.7 (2.0-20.8)	0.1565
IL2	15.7 (12.2–32.9)	16.0 (10.3–36.0)	0.683	17.8 (11.1–34.0)	14.5 (9.8–83.4)	0.881
IL6	21.1 (11.7–47.0)	10.0 (8.3–25.6)	**0.027**	10.4 (8.9–23.9)	10.0 (7.5–27.9)	0.084
IL7	15.5 (10.8–32.1)	18.1 (9.7–49.9)	0.982	16.8 (9.7–52.7)	18.1 (7.6–53.0)	0.943
IL8	4301 (2126–6997)^a^	2176 (1546–3325)	**0.037**	1812 (881.6–2430)^a^	2226 (1553–3502)	**0.037**
IL15	18.4 (13.5–33.4)	14.4 (11.0-34.7)	0.638	13.0 (11.4–26.4)	22.1 (10.8-126.2)	0.633
IP10	4.9 (4.2–5.2)	13.9 (4.2-181.7)	0.787	10.6 (4.1–89.3)	42.4 (4.2-260.2)	0.166
KC_like	461.7 (130.9-943.6)^a^	155.9 (89.1-291.4)	**0.014**	130.9 (52.1-320.7)^a^	178.4 (94.8-282.1)	**0.036**
IL10	13.8 (12.4–18.4)	13.8 (12.1–17.2)	0.787	13.5 (10.5–15.0)	14.5 (12.4–20.3)	0.469
IL18	11.1 (8.0-17.1)	10.8 (7.3–27.5)	0.764	8.3 (6.8–21.9)	13.7 (8.0-60.8)	0.253
MCP1	195.6 (138.9-432.2)^a^	108.4 (91.8-133.2)	**0.002**	103.2 (94.8-119.5)^a^	117.5 (91.8-212.6)	**0.005**
TNFα	14.3 (12.4–33.9)	14.3 (11.5–34.2)	0.638	12.9 (10.6–27.3)	18.2 (11.5–69.6)	0.581

P-values in bold highlight statistical significance between the groups

*Mann Whitney test assessing possible statistical differences between Diabetes mellitus (DM) and controls (total) groups; **Kruskal-Wallis test assessing possible statistical differences between Diabetes mellitus (DM) group and Control groups 1 and 2. Same letters indicate statistical significance (*P* < 0.05) between groups according to Dunn’s multiple comparison test

Salivary IL-15 and MCP1 were 1.3-fold higher in dogs with Diabetes mellitus compared to controls  (*P* < 0.05 and *P* < 0.01, respectively), while KC-like chemokine was 1.4-fold lower in dogs with DM as compared with controls (*P* < 0.01) (Table 3).

**Table 3:** Median (25–75 percentile) data of cytokines in saliva of dogs with Diabetes mellitus (*n* = 16) and controls (total, *n* = 45) with healthy gingiva (Control group 1 (CG1), *n* = 22) and with gingivitis (Control group 2 (CG2), *n* = 23)

	DM	Controls (total)	P*	CG1	CG2	P**
GM_CSF	14.2 (10.2–20.5)^a^	12.0 (9.3–15.3)	0.144	10.8 (6.5–13.5)^ab^	15.1 (10.4–19.1)^b^	**0.011**
IFNg	2.2 (1.2–3.2)	1.5 (1.0-2.4)	0.182	1.2 (0.8–2.4)	1.6 (1.2–2.6)	0.161
IL2	15.1 (10.7–20.5)	13.8 (9.6–19.0)	0.589	11.6 (8.0-15.6)	16.1 (11.6–23.4)	0.083
IL6	16.1 (11.0–25.0)^a^	11.5 (7.2–17.4)	0.097	9.5 (6.4–12.4)^ab^	14.0 (10.5–18.6)^a^	**0.011**
IL7	14.3 (10.6–26.8)	13.8 (8.7–19.5)	0.299	10.4 (6.1–14.0)^a^	16.5 (13.8–25.6)^a^	**0.006**
IL8	16,708 (12,495–26,982)	15,468 (10,859–19,280)	0.358	12,730 (8984–16,190)^a^	18,550 (14,897–26,708)^a^	**0.004**
IL15	15.4 (11.7–33.3)^a^	12.3 (7.8–15.7)	**0.028**	9.5 (6.1–12.3)^ab^	14.4 (10.9–24.4)^b^	**< 0.001**
IP10	4.3 (3.5–7.3)	5.5 (3.7–6.9)	0.617	4.0 (3.1–5.6)^a^	6.5 (4.3–10.0)^a^	**0.014**
KC_like	1884 (854.4–2175)^a^	2628 (1651–3626)	**0.004**	2255 (1395–3035)^b^	3482 (2416–4232)^ab^	**< 0.001**
IL10	16.6 (12.5–23.9)	15.5 (5.3–33.4)	0.529	8.5 (4.5–20.4)^a^	23.2 (11.8–43.9)^a^	**0.015**
IL18	9.2 (5.6–14.5)	7.4 (5.3–12.8)	0.550	6.7 (4.3–12.8)	10.0 (6.5–14.5)	0.310
MCP1	90.2 (54.5-108.9)^a^	69.8 (42.1–82.3)	**0.004**	59.6 (42.1–74.3)^ab^	74.3 (51.5-105.4)^b^	**< 0.001**
TNFα	23.7 (15.5–87.6)	24.1 (14.4–50.7)	0.487	15.5 (11.4–24.9)^a^	36.2 (20.3–60.9)^a^	**0.007**

P-values in bold highlight statistical significance between the groups

*Mann Whitney test; **Kruskal-Wallis test. Same letters indicate statistical significance (*P* < 0.05) between groups according to Dunn’s multiple comparison test

When control dogs were subdivided into two groups according to the health status of the gums, Kruskal-Wallis test followed by Dunn`s multiple comparison tests revealed statistically significantly higher levels of IL-8, KC-like and MCP1 in serum in CG1 dogs (healthy gingiva) compared to dogs with DM (Table 2). No other statistically significant differences were detected between the other groups of the study.

In case of saliva, Kruskal-Wallis test highlighted statistically significant differences between the two healthy groups in all evaluated cytokines, with the exception of IFNg, IL-2 and IL-18 (Table 3). According to Dunn`s multiple comparison test, dogs with DM presented statistically higher GM-CSF, IL6, IL15, and MCP1 levels as compared with CG1 (healthy gingiva), and lower KC-like chemokine as compared with CG2 (gingivitis). Finally, salivary levels GM-CSF, IL-6, IL-7, IL-15, IP-10, KC-like, IL-10, IL-18, MCP1, TNFα were statistically significantly higher in the saliva of CG2 in comparison with CG1.

## Discussion

The results of this study indicate that canine DM is related to changes in both serum and saliva pro-inflammatory cytokines. In the same line, using TMT-based approach, we have previously reported the presence of inflammatory status in dogs with DM (healthy gingiva) in relation to healthy dogs (healthy gingiva) [14]. However, the detection of pro-inflammatory status in canine DM could be blunted by the presence of gingivitis.

In serum, IL- 6, IL-8, KC-like and MCP1 were higher in dogs with DM than in control dogs. However, when control dogs were subdivided into two groups according to gingival health, no statistically significant changes were found in serum between dogs with DM and dogs with gingivitis which were otherwise healthy. These results in part agree with data reported by Ah Young Kim et al. [15], who did not detect statistically significant changes in serum IL-6, IL-10, IL-18 and TNFɑ of dogs with DM in comparison to healthy controls. However, unfortunately, they did not assess the oral health status of either group of dogs.

In saliva, when cytokine levels were compared between all control dogs and dogs with DM, two of them, namely IL-15 and MCP1, were at higher concentrations and one, KC-like chemokine, was at lower concentrations in saliva of DM dogs. However, when controls were subdivided into two groups according to oral health status, only KC-like chemokine showed differences between the group of dogs with DM and the dogs with gingivitis. This could be due to the effect of gingivitis that increase cytokine concentrations; since the presence of gingivitis in healthy dogs resulted in a higher number of cytokines altered in saliva (*n* = 10) than the presence of DM (*n* = 4) compared to dogs with healthy gums. Therefore, it can be stated that oral health is a main confounding factor for cytokine determination in saliva in DM in dogs.

Salivary KC-like chemokine was higher in the gingivitis group than in dogs without gingivitis or even DM. The increase in circulating levels of KC-like chemokine was previously reported in dogs with bacterial sepsis due to pyometra [16]. These data suggest that the increase in salivary KC-like chemokine levels observed in the present study in dogs with gingivitis could be due to the involvement of oral bacteria in the pathogenesis of this disease . However, further studies should be performed to clarify this topic.

The main limitations of the study were the relatively small number of animals used and, ideally, healthy dogs with gingivitis would have been further divided into different groups according to its severity . Furthermore, it would be important to assess possible changes in cytokines between dogs with DM with and without gingivitis. Therefore, the study should be considered as preliminary and should be confirmed in a larger population.

In conclusion, dogs with DM showed an increase in serum and salivary cytokines compared to healthy dogs. However, the oral health status shouldalways be considered, as the presence of gingivitis also alters cytokine levels in both biofluids, and could result in blunted differences between healthy dogs and dogs with DM.

## Data Availability

The datasets generated and/or analysed during the current study are not publicly available due to ethical and data protection reasons but are available from the corresponding author on reasonable request.
